# Cross-platform assessment of short-form video quality on the gut–liver axis: informational integrity and engagement disparity

**DOI:** 10.3389/fcimb.2025.1732375

**Published:** 2025-12-10

**Authors:** Man Sun, Dan Zang, Jun Chen

**Affiliations:** Department of Oncology, The Second Hospital of Dalian Medical University, Dalian, Liaoning, China

**Keywords:** gut microbiota, liver disease, gut-liver axis, health information quality, short-form videos, social media platforms

## Abstract

**Objective:**

The gut–liver axis has emerged as a pivotal focus in hepatology and metabolic disease research. However, the quality of public-facing health information, particularly in short-form video content, remains largely unexamined.

**Methods:**

Between January 2021 and October 2025, we systematically screened and analyzed 210 short videos (70 per platform) on the gut–liver axis. Basic metadata were extracted, and video quality was assessed using three validated tools: the modified DISCERN instrument, JAMA Benchmark Criteria, and Global Quality Score (GQS). Pearson correlation was used to explore associations between video metrics and quality scores.

**Results:**

Bilibili videos showed the highest educational quality (mean GQS: 3.79), while TikTok videos had greater engagement (median likes: 74.00). Videos uploaded by healthcare professionals scored significantly higher across all quality measures (SUM score: 9.03 *vs* 3.87, *p* < 0.001). No significant correlation was found between engagement metrics and content quality.

**Conclusion:**

A misalignment exists between user engagement and informational quality in gut–liver-related short videos. Content from verified health professionals delivers superior educational value yet remains algorithmically underprioritized. Efforts to enhance digital health communication should focus on promoting expert-led content, verifying source credentials, and integrating quality-weighted algorithms.

## 1 Introduction

The gut–liver axis has evolved from a mechanistic framework to a translational paradigm at the intersection of microbiology, immunology, and hepatology (Kim et al., 2024; Li et al., 2025). This bidirectional interface—linking microbial metabolism, mucosal immunity, and hepatic signaling—has redefined the pathophysiology of chronic liver diseases and catalyzed new therapeutic frontiers, from dietary modulation to fecal microbiota transplantation (FMT) (Tilg et al., 2022). However, as public interest rises, the translation of these scientific concepts into online health information often occurs without systematic oversight, increasing the risk of oversimplification and misinterpretation (Porcari et al., 2023).

Short-video platforms such as TikTok, Bilibili, and Xiaohongshu now constitute the world’s fastest-growing channels of biomedical diffusion, democratizing access to information while simultaneously eroding the boundary between expert knowledge and popular belief (Qin et al., 2024; (Monroe) Meng et al., 2024). Algorithmic optimization often prioritizes virality over veracity, elevating engagement metrics at the expense of informational rigor (Metzler and Garcia, 2024). In the context of microbiome-based therapies—particularly FMT, which traverses experimental, ethical, and regulatory domains—this dynamic is not merely a communication concern but a potential vector for clinical misinformation and behavioral risk (Hou et al., 2025).

Importantly, the gut–liver axis is especially prone to consumer-level distortion. Under the banner of “detox,” “microbiome resetting,” or “liver cleansing,” this scientific construct is frequently co-opted by marketers of functional beverages, probiotic supplements, and herbal remedies—often stripped of mechanistic accuracy or clinical grounding (Anand and Mande, 2022; Strand, 2022). These narratives borrow scientific legitimacy while bypassing regulatory scrutiny, contributing to overclaimed health benefits and microbiome-related misinformation. These trends highlight the need to evaluate how gut–liver–related information is presented within high-traffic digital ecosystems.

Despite the exponential growth of digital health research, limited attention has been paid to how algorithmic platforms translate scientific complexity into lay perceptions—or how engagement metrics such as likes, shares, and comments may inflate the perceived credibility of content regardless of its accuracy (De Santis et al., 2023). The gut–liver axis, situated at the intersection of translational science and online discourse, offers a compelling lens to interrogate this disconnect between digital visibility and biomedical validity (Tripathi et al., 2018; Lin et al., 2025). Yet no study has systematically assessed the quality, reliability, or platform-specific differences of short-form video content on this topic, representing a notable research gap.

To address this epistemic and ethical gap, we conducted a cross-platform, quality-of-information audit of short videos related to gut microbiota and liver disease. Using validated instruments (mDISCERN, JAMA benchmarks, GQS) alongside engagement-quality correlation analyses, we aimed to evaluate: (i) the overall quality and reliability of gut–liver axis content across major short-video platforms; (ii) differences among TikTok, Bilibili, and Xiaohongshu—three platforms with distinct content norms, audience demographics, and creator ecosystems; and (iii) whether engagement metrics align with or diverge from educational value (Mohammadzadeh Azarabadi et al., 2025). By clarifying these dimensions, the study provides empirical evidence to guide digital-health governance, expert participation, and public-facing microbiome communication.

## 2 Materials and methods

### 2.1 Video collection

This cross-sectional descriptive study analyzed publicly available science popularization videos addressing gut microbiota in the context of liver disease management. Three major short-form video platforms—TikTok, Bilibili, and Xiaohongshu—were selected to represent both global and region-specific digital health communication environments. Video retrieval was conducted from January 2021 to October 2025.

To reflect real-world user behavior, we searched each platform using commonly used Chinese-language terms related to “gut microbiota,” “liver disease,” and the “gut–liver axis,” including both medical phrases and everyday expressions. All searches were conducted in a logged-out browser with cleared cache and history to avoid algorithm-driven content filtering. Videos were collected based on the platform’s default recommendation logic—typically sorted by relevance or popularity. Given the linguistic composition of the user base and the dominance of Chinese-language content on these platforms, only Mandarin-language searches were included in the present study.

Recently published videos (within two weeks of retrieval), advertisements, duplicates, and irrelevant content were excluded to ensure content stability. The first 70 eligible videos per platform were included for analysis. For each video, metadata were extracted, including: title, URL, number of likes, comments, and shares, upload date, duration, days since upload, primary content focus, video source (such as professional institutions, or non-Professional institutions), and view count (available on Bilibili only).

### 2.2 Video quality assessments

To evaluate the reliability and overall quality of the included videos, three validated instruments were utilized: the Journal of the American Medical Association (JAMA) Benchmark Criteria, the Global Quality Score (GQS), and the modified DISCERN instrument (Charnock et al., 1999; Azer, 2020; Kyarunts et al., 2022). These tools have been widely applied in previous studies assessing online health information quality. Detailed scoring criteria for each tool are provided in **Supplementary Table 1**.

To avoid terminological ambiguity, we operationalized “video quality” and “content quality” as a multidimensional construct comprising structural rigor, source credibility, and evidence-orientation. Structural rigor (clarity, coherence, organization, citation transparency) was assessed through GQS and the structural components of the modified DISCERN tool. Source credibility and informational reliability (authorship, attribution, disclosure, currency) were evaluated using the JAMA Benchmark Criteria and reliability-focused DISCERN items. Evidence-orientation (balanced presentation, reference to evidence, acknowledgment of uncertainty) was captured through DISCERN indicators. As these instruments evaluate information presentation rather than adjudicate scientific correctness, the composite SUM score reflects the overall educational and informational quality of each video rather than production aesthetics or factual precision.

The JAMA Benchmark Criteria evaluate source transparency and credibility across four domains: authorship, attribution, disclosure, and currency (Kloosterboer et al., 2019). Each satisfied domain is awarded one point, yielding a total score ranging from 0 to 4, with higher scores reflecting greater source reliability. The Global Quality Scale (GQS) uses a 5-point Likert scale to assess the overall flow, coherence, and educational value of the video content for a general audience (Kunze et al., 2020). A score of 1 indicates poor quality with minimal utility, while a score of 5 represents excellent quality with high informational value. The modified DISCERN tool, originally developed for evaluating written health information, was adapted for video assessment (Rees et al., 2002). In this study, the first section comprising five binary (yes/no) items was used to evaluate clarity of objectives, relevance of content, balance of information, source transparency, and discussion of uncertainty. Each affirmative response scored one point, resulting in a total score ranging from 0 to 5, with higher scores denoting greater reliability.

Two independent reviewers with medical training assessed each video after standardized training to reduce evaluation bias. Inter-rater reliability was quantified using Cohen’s kappa (κ). As per the Landis and Koch criteria, κ values > 0.8 indicate excellent agreement, 0.6–0.8 substantial, 0.4–0.6 moderate, and < 0.4 poor (Landis and Koch, 1977). Discrepancies were resolved through discussion until full consensus was reached.

### 2.3 Statistical analysis

All statistical analyses were performed using non-parametric tests due to non-normality of the data. Statistical analyses were conducted using the full dataset of 210 videos (n = 210). Descriptive statistics were presented as medians with interquartile ranges (IQR). The Mann–Whitney U test was used for two-group comparisons, while the Kruskal–Wallis H test was applied for comparisons involving three or more groups, followed by appropriate *post hoc* analysis with effect size estimation. Spearman’s rank correlation was used to examine associations between video metrics and quality scores. Inter-rater reliability was assessed using Cohen’s kappa statistic. A p-value of <0.05 was considered statistically significant. All analyses were performed using IBM SPSS Statistics version 25, and visualizations were generated using GraphPad Prism version 9.

## 3. Results

### 3.1 Video screening and selection

**Figure 1:**
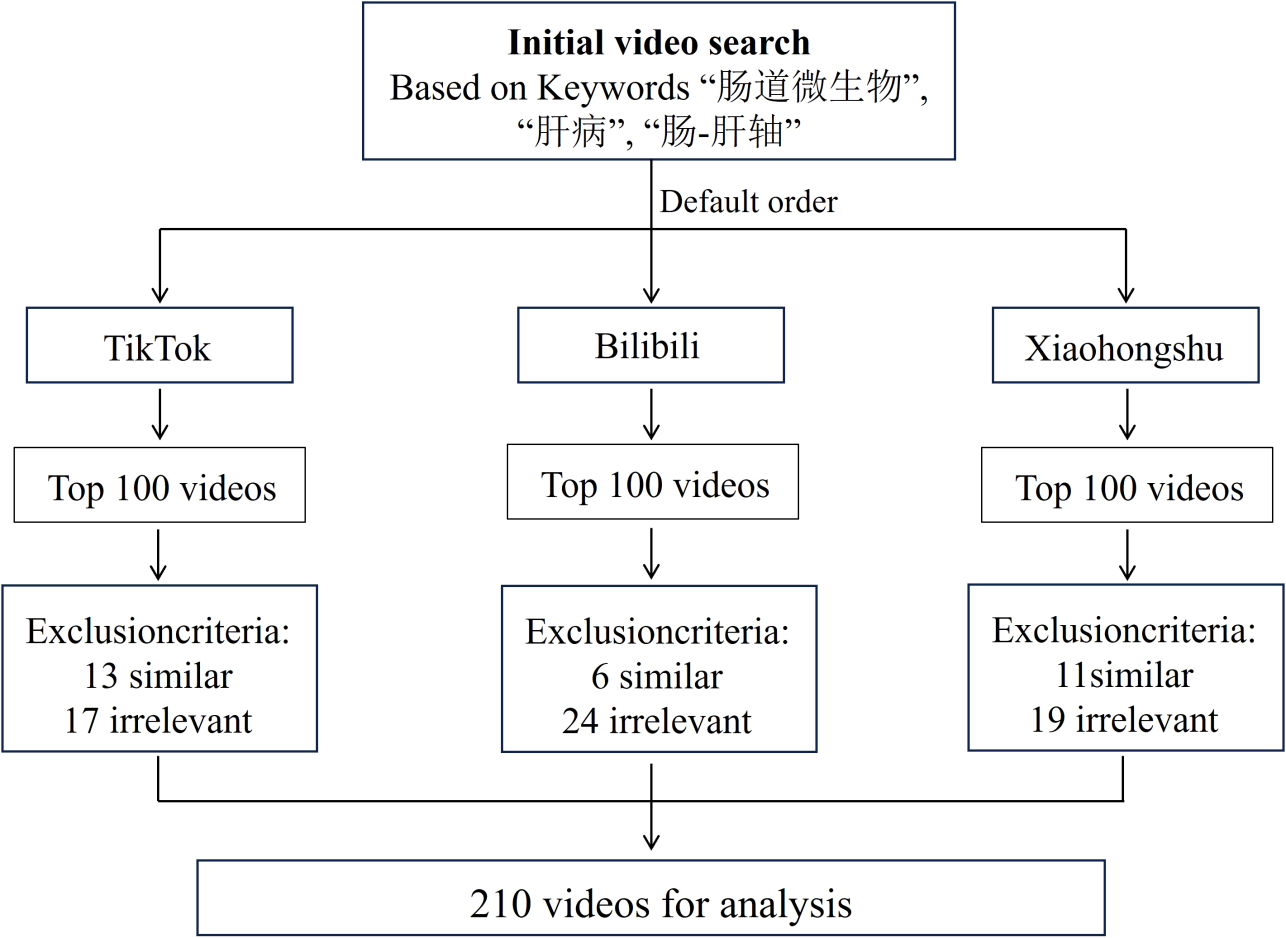
Flow diagram of video selection across three short-form video platforms.

Among the top 300 videos initially screened on each platform (Xiaohongshu, Bilibili, and TikTok), a total of 210 videos met the inclusion criteria and were retained for detailed analysis. **Figure 1** provides a schematic overview of the entire research workflow, including video retrieval, screening, and inclusion. In total, 690 videos were excluded during the screening process, including 432 that were irrelevant to gut microbiota or liver disease, 136 that were duplicates, 84 recorded in languages other than English or Chinese, and 38 that were advertisements or unavailable at the time of assessment. The final dataset comprised 70 videos from each platform, which were subsequently evaluated using the JAMA, GQS, and modified DISCERN instruments. **Supplementary Table 2** provides the full breakdown of inclusion and scoring criteria.

### 3.2 Descriptive characteristics of included videos

A total of 210 short-form popular science videos were analyzed, with an equal number (n=70) selected from Bilibili, TikTok, and Xiaohongshu. Across all four quality assessment tools, Bilibili videos demonstrated significantly higher scores, including the modified DISCERN (3.33 ± 0.99), SUM (9.86 ± 2.67), JAMA (2.74 ± 0.90), and Global Quality Score (3.79 ± 1.08), all with p-values < 0.001. In contrast, TikTok videos achieved the highest levels of user engagement, reflected by a greater number of likes (median 74.00), comments (median 9.50), and shares (median 20.00), compared to the other two platforms (all p < 0.001). Notably, Bilibili videos had the longest duration (median 478 seconds), whereas videos from TikTok and Xiaohongshu were significantly shorter (66 and 77 seconds, respectively). These findings suggest a trade-off between educational quality and audience engagement among platforms. The median number of lengths, views, comments, likes, shares, followers/subscribers and other baseline information are reported in **Table 1**. There were statistically significant variations in videographic characteristics among the social platforms.

**Table 1:** Cross-platform comparison of video quality scores (mDISCERN, JAMA, GQS) and engagement metrics across TikTok, Bilibili, and Xiaohongshu.

Variables	Total (n = 210)	Bilibili (n = 70)	TikTok (n = 70)	Xiaohongshu (n = 70)	Statistic	*P*
M Dis Score, Mean ± SD	2.74 ± 1.16	3.33 ± 0.99	2.54 ± 1.10	2.36 ± 1.17	F=15.73	**<0.001**
SUM score, Mean ± SD	8.29 ± 2.86	9.86 ± 2.67	7.89 ± 2.43	7.13 ± 2.79	F=20.03	**<0.001**
JAMA score, Mean ± SD	2.28 ± 0.88	2.74 ± 0.90	2.13 ± 0.74	1.97 ± 0.80	F=17.56	**<0.001**
GQS score, Mean ± SD	3.27 ± 1.11	3.79 ± 1.08	3.21 ± 0.96	2.80 ± 1.08	F=15.79	**<0.001**
Likes, M (Q_1_, Q_3_)	17.00 (4.00, 93.00)	21.00 (4.00,119.00)	74.00 (35.00,223.50)	4.00 (1.00,10.75)	χ²=73.52#	**<0.001**
Comments, M (Q_1_, Q_3_)	1.00 (0.00, 7.75)	0.00 (0.00,2.75)	9.50 (4.00,39.50)	0.00 (0.00,1.00)	χ²=87.93#	**<0.001**
Favorites, M (Q_1_, Q_3_)	10.50 (2.00, 55.00)	48.50 (4.00,187.25)	18.50 (8.00,102.75)	2.00 (0.00,8.75)	χ²=50.76#	**<0.001**
Shares, M (Q_1_, Q_3_)	8.00 (1.00, 35.75)	14.00 (1.00,50.50)	20.00 (4.00,129.00)	2.00 (0.00,7.00)	χ²=40.25#	**<0.001**
Duration, M (Q_1_, Q_3_)	94.50 (58.00, 251.25)	478.00 (141.00,1759.00)	66.00 (53.00,98.75)	77.00 (54.25,114.25)	χ²=72.17#	**<0.001**

F: ANOVA, #: Kruskal-waills test.

SD, standard deviation; M, Median; Q_1_: 1st Quartile, Q_3_: 3st Quartile.

The bold values indicate statistically significant results (p < 0.05).

### 3.3 Author characteristics

**Figure 2:**
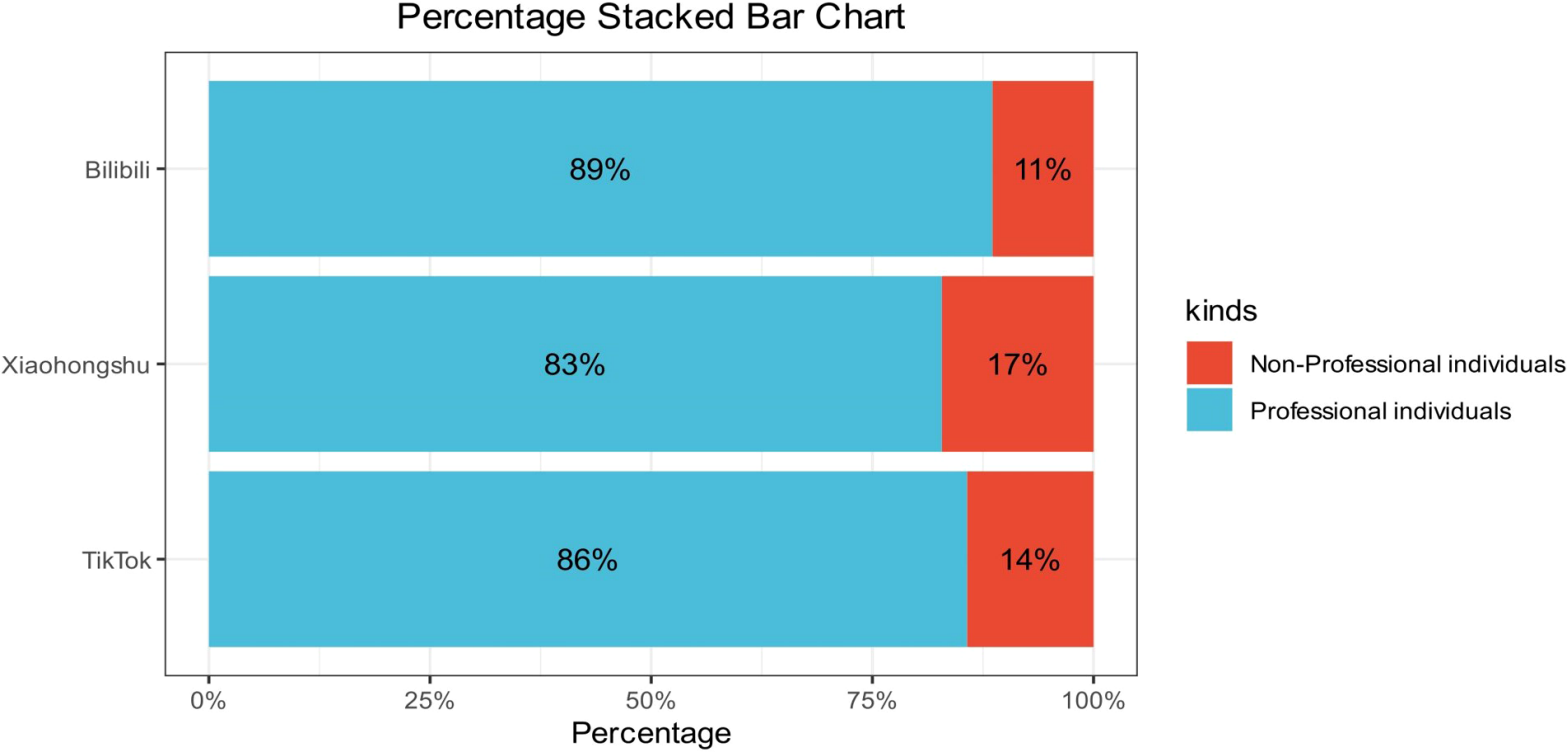
Distribution of professional and non-professional content creators across platforms.

Among the 210 videos analyzed, the majority were uploaded by individuals with professional backgrounds across all three platforms. Bilibili demonstrated the highest proportion of professionally affiliated creators (89%), followed by TikTok (86%) and Xiaohongshu (83%). In contrast, non-professional contributors accounted for 11% of Bilibili videos, 14% of TikTok videos, and 17% of Xiaohongshu videos (**Figure 2**). These differences may reflect platform-specific audience demographics and content ecosystem characteristics, with Bilibili being more academically oriented and Xiaohongshu featuring more lifestyle and consumer-generated content.

To further evaluate the influence of author background on video quality and user engagement, we compared professionally affiliated and non-professional content creators across multiple assessment metrics. Videos produced by professionals (n = 180) consistently outperformed those from non-professional sources (n = 30) in all quality domains, including mDISCERN (3.01 ± 0.98 vs. 1.17 ± 0.87), SUM score (9.03 ± 2.31 vs. 3.87 ± 1.59), JAMA (2.45 ± 0.81 vs. 1.27 ± 0.45), and GQS (3.57 ± 0.85 vs. 1.43 ± 0.63), with all differences reaching statistical significance (p<0.001). In terms of user engagement, professionally authored videos also received significantly more likes (median: 23.00 vs. 6.50; p = 0.015), favorites (13.00 vs. 8.00; p = 0.013), and had longer durations (98.00 vs. 66.50 seconds; p = 0.010). However, no significant differences were observed in the number of comments (p = 0.223) or shares (p = 0.091) between the two groups (**Table 2**). These findings underscore the strong association between author expertise and both informational credibility and partial indicators of audience receptivity.

**Table 2:** Comparison of video quality scores and engagement metrics between professional and non-professional content creators across the three platforms.

Variables	Total (n = 210)	Non-professional institutions (n = 30)	Professional institutions (n = 180)	Statistic	*P*
M Dis Score, Mean ± SD	2.74 ± 1.16	1.17 ± 0.87	3.01 ± 0.98	t=-9.63	**<.001**
SUM score, Mean ± SD	8.29 ± 2.86	3.87 ± 1.59	9.03 ± 2.31	t=-15.28	**<0.001**
JAMA score, Mean ± SD	2.28 ± 0.88	1.27 ± 0.45	2.45 ± 0.81	t=-11.59	**<0.001**
GQS score, Mean ± SD	3.27 ± 1.11	1.43 ± 0.63	3.57 ± 0.85	t=-13.15	**<0.001**
Likes, M (Q_1_, Q_3_)	17.00 (4.00, 93.00)	6.50 (3.00, 20.50)	23.00 (4.50, 102.00)	Z=-2.43	**0.015**
Comments, M (Q_1_, Q_3_)	1.00 (0.00, 7.75)	0.00 (0.00, 3.75)	2.00 (0.00, 8.25)	Z=-1.22	0.223
Favorites, M (Q_1_, Q_3_)	10.50 (2.00, 55.00)	8.00 (1.00, 16.00)	13.00 (2.00, 65.50)	Z=-2.47	**0.013**
Shares, M (Q_1_, Q_3_)	8.00 (1.00, 35.75)	4.00 (1.00, 18.50)	10.00 (1.00, 36.25)	Z=-1.69	0.091
Duration, M (Q_1_, Q_3_)	94.50 (58.00, 251.25)	66.50 (52.75, 102.50)	98.00 (61.00, 288.50)	Z=-2.56	**0.01**

t, t-test; Z, Mann-Whitney test.

SD, standard deviation; M, Median; Q_1_, 1st Quartile; Q_3_, 3st Quartile.

The bold values indicate statistically significant results (p < 0.05).

### 3.4 Video quality and reliability assessment

#### 3.4.1 Inter-rater agreement

To ensure reliability and minimize subjective bias in video quality assessment, two independent coders with medical training evaluated each video using standardized scoring instruments. Inter-rater agreement was quantified using Cohen’s kappa coefficient, yielding a value of 0.913—indicative of almost perfect agreement according to the classification by Landis and Koch. As shown in the confusion matrix (**Table 3**), 35 out of 36 videos received a score of “3” from both raters, and full concordance was observed for all videos rated “5.” Minor discrepancies occurred primarily between adjacent categories, further supporting the robustness and reproducibility of the evaluation process.

**Table 3:** Confusion matrix of inter-rater agreement in video quality scoring and Cohen’s Kappa coefficient.

Cohen’s Kappa coefficient = 0.913						
Coder A	Coder B					SUM
	3	4	5	2	1	
3	35	0	0	1	0	36
4	1	17	0	0	0	18
5	0	0	6	0	0	6
2	0	0	0	3	1	4
1	0	0	0	1	5	6
SUM	36	17	6	5	6	70

#### 3.4.2 Video quality across platforms

**Figure 3:**
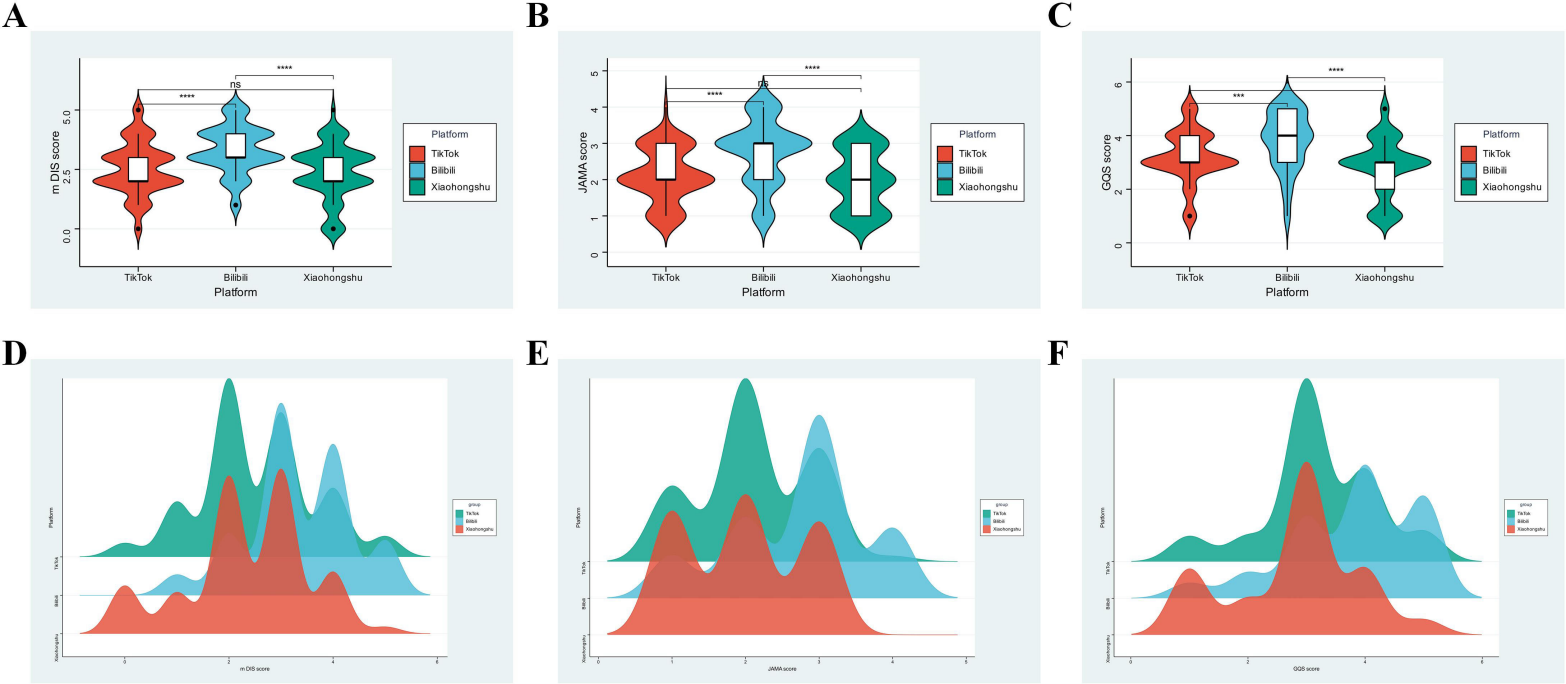
Cross-platform comparison of video quality metrics and subdomain scores. **(A–C)** Violin plots showing the distribution of mDISCERN, JAMA, and GQS scores across TikTok, Bilibili, and Xiaohongshu. Statistical comparisons were performed using the Kruskal–Wallis test followed by Dunn’s *post hoc* test. **(D–F)** Ridge density plots for each scoring tool, illustrating platform-specific score concentration and heterogeneity. *** indicates p < 0.001; **** indicates p < 0.0001.

We assessed video quality across platforms using three validated instruments: the modified DISCERN (mDISCERN), JAMA benchmark criteria, and Global Quality Score (GQS). As shown in **Figures 3A–C**, Bilibili consistently achieved the highest median scores across all three metrics, followed by Xiaohongshu and TikTok. The violin plots visualize central tendency and distributional shape, where wider sections indicate higher density of observations.

Statistical comparisons confirmed these platform-level differences. For mDISCERN, Bilibili significantly outperformed TikTok (*p* < 0.0001) and Xiaohongshu (*p* < 0.0001), with TikTok also scoring higher than Xiaohongshu (*p* < 0.0001). A similar trend was observed in JAMA scores, where Bilibili surpassed both TikTok and Xiaohongshu (*p* < 0.0001 for both comparisons), while no significant difference was found between the latter two (*p* > 0.05). For GQS, Bilibili again led (*p* < 0.001 *vs*. TikTok; *p* < 0.0001 *vs*. Xiaohongshu), whereas TikTok and Xiaohongshu did not differ significantly (*p* > 0.05). These findings suggest that Bilibili consistently provides higher-quality educational content on gut microbiota and liver disease.

To further explore score concentration and distributional characteristics, we employed ridge density plots (**Figures 3D–F**), which reinforced the violin plot findings. For mDISCERN, Bilibili exhibited a unimodal distribution peaking between scores 3–5, indicative of generally high-quality content. In contrast, TikTok and Xiaohongshu showed more dispersed distributions, with peaks concentrated in the 1–3 score range and noticeable tails extending toward lower scores.

The JAMA score distributions mirrored these patterns. Bilibili’s density peaked between 2.5–4, indicating reliable fulfillment of authorship, attribution, disclosure, and currency standards. TikTok and Xiaohongshu scores concentrated between 1–3, suggestive of partial or inconsistent compliance. GQS plots similarly showed Bilibili clustering around high scores (Tilg et al., 2022; Porcari et al., 2023; (Monroe) Meng et al., 2024), while the other two platforms skewed toward lower scores (Tilg et al., 2022; Li et al., 2025), with more dispersed distributions.

**Figure 4:**
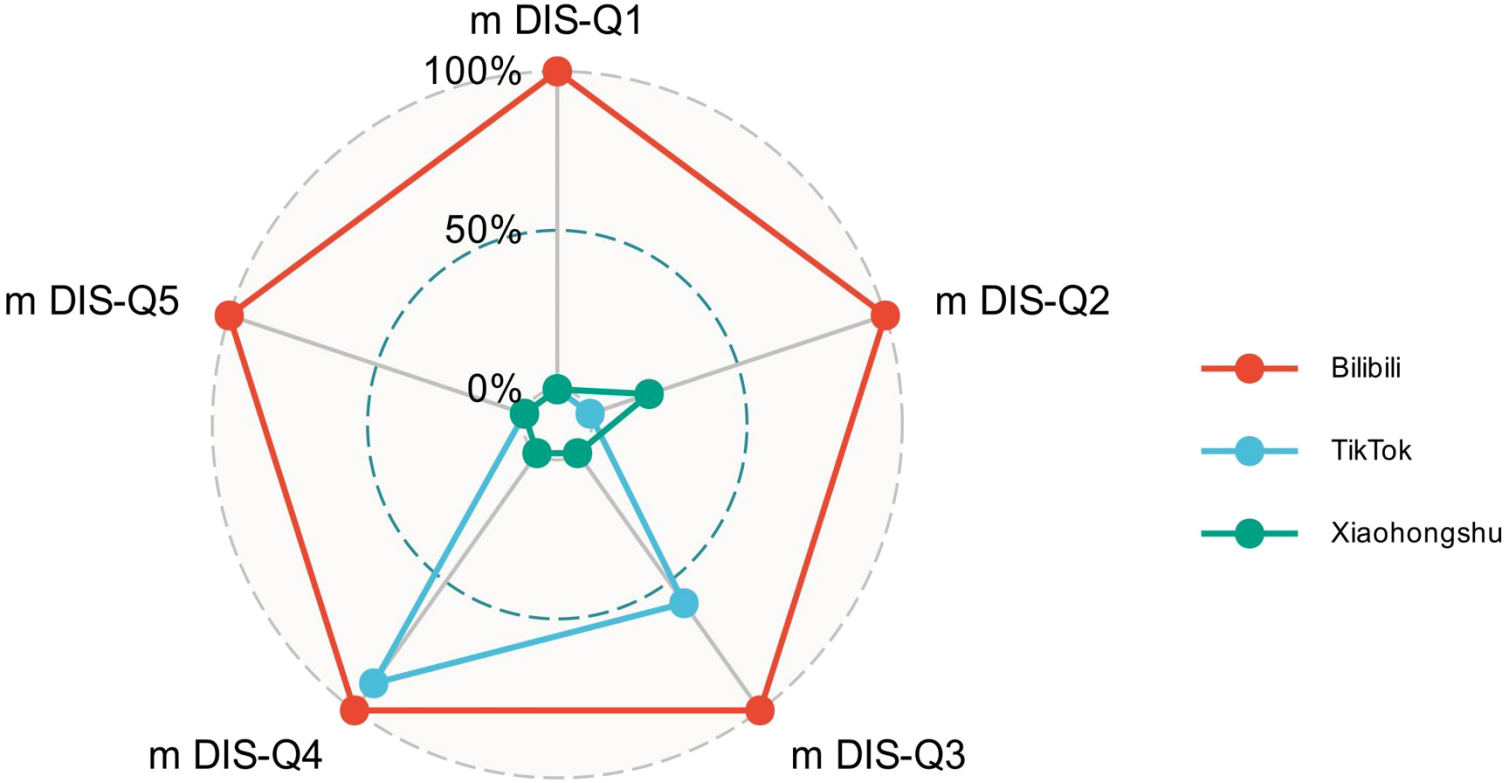
Radar chart showing the mDISCERN subdomain scores (Q1–Q5) across three platforms.

To gain deeper insights into how different aspects of quality contributed to platform-level variation, we analyzed mDISCERN subdomain scores (Q1–Q5), as visualized in **Figure 4**. Bilibili exhibited a balanced and near-maximal profile across all five domains—clarity of aims (Q1), source citation (Q2), content relevance (Q3), information reliability (Q4), and discussion of uncertainty (Q5). In contrast, TikTok and Xiaohongshu scored consistently lower across all domains, with particularly poor performance on Q2 and Q5. These results suggest that Bilibili videos are more likely to cite credible sources and acknowledge content limitations, whereas the other two platforms exhibit fragmentary and informationally diluted presentations.

Overall, both visual and statistical analyses consistently reveal Bilibili’s advantage in video quality under all three scoring frameworks. Compared to TikTok and Xiaohongshu, Bilibili not only achieved higher average scores, but also demonstrated tighter distributions and stronger compliance with quality standards.

### 3.5 Correlation analysis

To elucidate the relationship between user engagement and video quality, we conducted a Pearson correlation analysis encompassing six key variables: likes, comments, shares, favorites, video duration (in seconds), and the composite SUM quality score (an aggregate of mDISCERN, JAMA, and GQS).

As presented in **Table 4**, the four core engagement indicators—likes, comments, shares, and favorites—demonstrated extremely high intercorrelations, all statistically significant at the p < 0.01 level. Specifically, likes were nearly perfectly correlated with comments (r = 0.999), shares (r = 0.998), and favorites (r = 0.992), indicating strong collinearity and suggesting that these metrics tend to co-occur across platforms regardless of content substance.

**Table 4:** Pearson correlation matrix among user engagement metrics and overall video quality (N = 210).

Correlation							
		Likes	Comments	Shares	Favorites	Duration	SUM score
Likes	Pearson Correlation	1	0.999**	0.998**	0.992**	-0.016	0.023
	Sig. (2-tailed)		0.000	0.000	0.000	0.822	0.744
	Number of Cases	209	209	209	209	209	209
Comments	Pearson Correlation	0.999**	1	1.000**	0.988**	-0.012	0.022
	Sig. (2-tailed)	0.000		0.000	0.000	0.860	0.749
	Number of Cases	209	210	210	210	210	210
Shares	Pearson Correlation	0.988**	1.000**	1	0.986**	-0.017	0.018
	Sig. (2-tailed)	0.000	0.000		0.000	0.803	0.794
	Number of Cases	209	210	210	210	210	210
Favorites	Pearson Correlation	0.992**	0.988**	0.986**	1	0.046	0.063
	Sig. (2-tailed)	0.000	0.000	0.000		0.508	0.362
	Number of Cases	209	210	210	210	210	210
Duration	Pearson Correlation	-0.016	-0.012	-0.017	0.046	1	0.335**
	Sig. (2-tailed)	0.822	0.860	0.803	0.508		0.000
	Number of Cases	209	210	210	210	210	210
SUM score	Pearson Correlation	0.023	0.022	0.018	0.063	0.335**	1
	Sig. (2-tailed)	0.744	0.749	0.794	0.362	0.000	
	Number of Cases	209	210	210	210	210	210

**p < 0.01 (two-tailed) indicates statistical significance.

In contrast, video duration exhibited no meaningful correlation with likes (r = –0.016, p = 0.822), comments (r = –0.012, p = 0.860), shares (r = –0.017, p = 0.803), or favorites (r = 0.046, p = 0.508). This indicates that longer videos do not inherently drive greater engagement, underscoring the platform-specific nature of attention dynamics.

Crucially, the SUM video quality score showed no significant association with any of the engagement metrics: likes (r = 0.023, p = 0.744), comments (r = 0.022, p = 0.749), shares (r = 0.018, p = 0.794), or favorites (r = 0.063, p = 0.362). This disconnect suggests that content popularity may not reflect educational merit.

Notably, a moderate positive correlation was observed between video duration and quality score (r = 0.335, p < 0.01), implying that longer videos tend to feature more comprehensive or higher-quality information, potentially due to greater capacity for contextual elaboration or source citation.

Collectively, these findings reveal a misalignment between user engagement and educational value. High interaction metrics, while reflective of visibility or entertainment value, should not be misinterpreted as indicators of informational quality in the context of health communication on short-video platforms.

## 4 Discussion

Building on the statistical differences identified across the three platforms, with Bilibili showing consistently higher informational quality and TikTok generating substantially greater user engagement, our findings highlight a persistent disconnect between visibility and content quality. Our findings highlight a persistent disconnect between visibility and content quality in gut–liver axis–related short-form videos. Despite TikTok’s high engagement, it consistently ranked lowest across all educational indices. In contrast, Bilibili videos—characterized by longer duration and expert-driven production—achieved the highest quality scores (all p < 0.001) (Sun et al., 2025). Notably, engagement metrics showed no correlation with content quality, reinforcing that virality does not equal scientific rigor. The only content feature moderately associated with higher quality was video duration (r = 0.335, p < 0.01), suggesting that longer videos offer more room for context and citation—an often-overlooked factor on short-form platforms (van Kessel et al., 2022).

Subgroup analysis underscored the pivotal role of creator expertise in shaping both content quality and audience response. Videos produced by healthcare professionals or affiliated institutions consistently outperformed those from non-professionals across all evaluated domains, with average SUM scores of 9.03 versus 3.87, respectively (p < 0.001). Professionally authored videos also achieved higher levels of user approval, including likes and favorites, contradicting the notion that evidence-based content is less appealing (Kong et al., 2021). These findings refute the assumption that evidence-based content lacks appeal and underscore the dual value of professional contributors in ensuring both scientific accuracy and audience receptivity—particularly vital for complex topics like the gut–liver axis, where simplification may distort clinical understanding.

For instance, fecal microbiota transplantation (FMT), still under clinical evaluation for most liver-related conditions, is often misrepresented in short videos as a general “gut detox” tool—ignoring regulatory constraints, donor-screening protocols, and infection risks (Ma et al., 2025). Similarly, over-the-counter probiotics and so-called “gut-liver detox teas” are frequently marketed as cure-alls, supported by vague microbiome rhetoric unbacked by clinical evidence (Pan et al., 2025). This commodification of microbiome therapies undermines informed decision-making and may delay proper treatment.

Our findings challenge the presumed link between popularity and educational value. While engagement indicators—likes, comments, shares, and favorites—were highly intercorrelated, none aligned with video quality scores (Bortolotti, 2016). This suggests that algorithmic amplification often rewards emotional appeal over scientific substance. In many short-video ecosystems, algorithmic amplification prioritizes emotionally appealing or entertainment-oriented content, which suppresses informational depth by rewarding brief and attention-driven viewer responses. This dynamic tends to elevate highly engaging but educationally limited material. Longer videos function as an exception because their extended duration allows creators to provide context, incorporate evidence, and construct more coherent explanations. This structural flexibility helps clarify the moderate positive association between video duration and educational quality observed in our analysis, despite the broader algorithmic tendency to favor immediacy over informational rigor. Notably, longer duration emerged as the only quantitative predictor of quality, reinforcing the importance of temporal space for conveying complex health information (Mercer Moss et al., 2016).

Platform-level differences in user demographics and engagement behaviors may also influence visibility patterns. Social media platforms vary widely in terms of user base size, activity level, age distribution, educational background, and professional orientation. These factors may shape algorithmic amplification by determining which types of content generate rapid engagement signals. For example, platforms with younger or entertainment-oriented users may preferentially circulate visually stimulating short videos, whereas platforms with more academically oriented users may better support higher-quality educational content. Although user characteristics were not the primary focus of this study, acknowledging these differences provides important context for interpreting cross-platform disparities.

Correcting this imbalance requires coordinated interventions. At the creator level, incentives such as educational grants, CME credit, or institutional branding could encourage expert participation. Platforms should embed verifiable creator credentials—through medical badges, institutional tags, or tiered quality labels—and evolve algorithms to incorporate quality-weighted amplification. Recommendation systems designed to “serve societal good” have argued for integrating long-term value and content fidelity into feed logic (Lasser and Poechhacker, 2025; Milli et al., 2025). In addition, scalable safeguards—such as real-time peer-review tagging systems analogous to academic article review—may provide a viable path to maintain diversity without sacrificing reliability.

These efforts should be coupled with user-focused strategies to bolster digital health literacy. Campaigns addressing cognitive biases, trust heuristics, and information hygiene can improve public resilience against misinformation (Yuen et al., 2024; Ziapour et al., 2024). This is particularly urgent in microbiome and metabolic health, where patient decisions hinge on accurate understanding of multisystemic interactions. To our knowledge, this is the first multi-dimensional, cross-platform audit of gut–liver axis–related science videos using validated quality instruments. Unlike prior studies focusing on a single platform or disease, our work offers a broader lens into how platform dynamics, creator identity, and algorithmic visibility co-shape digital health narratives. These insights hold direct implications for hepatology, microbiome-based interventions, and translational education—domains where public understanding is critical to clinical uptake (Schweitzer et al., 2025).

Nonetheless, some limitations should be acknowledged. First, the cross-sectional design captures only a single timepoint, making it difficult to assess how content quality may evolve over time or in response to policy changes. Second, although we used validated scoring tools and ensured dual independent review, some degree of subjectivity is inevitable in quality assessment (Gijsen et al., 2020). Third, the study included only Chinese-language content from three major platforms (TikTok, Bilibili, and Xiaohongshu) and did not incorporate other ecosystems such as WeChat Video Accounts or YouTube, which may operate under different governance structures and user demographics. Fourth, all data reflect platform behavior as of October 2025; subsequent algorithmic updates may influence content visibility, engagement dynamics, and creator practices. Finally, cross-linguistic and cross-cultural variations were not evaluated, which may limit the generalizability of our findings to regions beyond the Mandarin-speaking context. Future research incorporating multilingual and cross-cultural analyses would provide a more comprehensive understanding of these dynamics.

Looking ahead, future research could explore several promising directions. AI-based tools may help detect misinformation and track semantic drift in real time. Longitudinal studies could shed light on how platform-level content changes in response to shifting algorithms or public health events. In addition, experimental studies might evaluate whether expert verification, visible credibility labels, or algorithm-level nudges can improve the visibility and trustworthiness of high-quality content. Ultimately, interdisciplinary collaboration—among clinicians, data scientists, platform designers, and regulators—will be key to aligning online health information ecosystems with public health priorities.

Beyond the practical considerations of platform design and governance, our findings point to a deeper epistemic challenge that shapes scientific communication in algorithmic environments. When relevance and visibility are driven by engagement metrics rather than informational rigor, public understanding of complex biomedical concepts becomes filtered through attention-oriented curation. This process not only influences what audiences encounter but also affects how scientific knowledge is prioritized, simplified, or marginalized within digital spaces. Addressing these epistemic distortions will require interdisciplinary collaboration to ensure that insights related to the gut–liver axis and biomedical science more broadly can circulate in a manner that preserves complexity, contextual integrity, and translational relevance.

## 5 Conclusion

In conclusion, this cross-platform analysis demonstrates a clear disconnect between the popularity and educational quality of short-form videos related to the gut–liver axis. While content from healthcare professionals consistently exhibited higher informational value, engagement metrics failed to reflect this, highlighting the limitations of algorithm-driven visibility. To improve digital health communication, platforms should prioritize content quality through expert verification and algorithmic adjustments, while parallel efforts to enhance public digital literacy are crucial for empowering evidence-based health decisions in the digital age.

## Data Availability

The original contributions presented in the study are included in the article/**Supplementary Material**. Further inquiries can be directed to the corresponding author.
