# Supplementary material for: Cross-platform assessment of short-form video quality on the gut–liver axis: informational integrity and engagement disparity
Source: Front Cell Infect Microbiol. 2025 Dec 10;15:1732375. doi: 10.3389/fcimb.2025.1732375 (PMC12727577; doi:10.3389/fcimb.2025.1732375)
Supplement: Supplementary file 1 [file Table1.docx]

**Supplementary scales**

Modified DISCERN quality criteria for assessing the reliability of video. (1 point for answer ‘yes’, 0 point for answer ‘no’)

| Reliability Score |
| --- |
| 1. Is the video clear, concise, and understandable? |
| 2. Are valid sources cited? |
| 3. Is the content presented balanced and unbiased? |
| 4. Are additional sources of content listed for patient reference? |
| 5. Are areas of uncertainty mentioned? |

Global Quality Score (GQS) (Scoring ranges from 1 to 5)

| GQS Definition | Score |
| --- | --- |
| Poor quality：Specifically, the content is illogical, the mobility is poor, most of the information is missing, and it is useless for patients. | 1 |
| Generally poor quality ：the content logic is poor, although some information is listed, more important information is still missing, and the use of patients is very limited. | 2 |
| Moderate quality：some important information is adequately discussed. | 3 |
| Good quality and flow：Specifically, the video logic is clear and smooth, covering most of the relevant information, which is useful for patients. | 4 |
| Excellent quality and flow：Specifically, the video logic is clear, and the content is very smooth, which is very useful for patients. | 5 |

The Journal of the American Medical Association (JAMA) benchmark criteria.

| Score* | Score component | |
| --- | --- | --- |
| 1 score | Authorship | Author and contributor credentials and their affiliations should be provided. |
| 1 score | Attribution | Clearly lists all copyright information and states references and sources for content. |
| 1 score | Currency | Initial date of posted content and subsequent updates to content should be provided. |
| 1 score | Disclosure | Conflicts of interest, funding, sponsorship, advertising, support, and video ownership  should be fully disclosed. |

*The criteria of each aspect were scored separately, and 1 point for each criterion with a total score of 4 points.
